# Clinical implications of non-breast cancer related findings on FDG-PET/CT scan prior to neoadjuvant chemotherapy in patients with breast cancer

**DOI:** 10.1007/s10549-024-07331-9

**Published:** 2024-06-12

**Authors:** Josefien P. van Olmen, A. Marjolein Schrijver, Marcel P. M. Stokkel, Claudette E. Loo, Jetske L. B. Gunster, Marie-Jeanne T. F. D. Vrancken Peeters, Frederieke H. van Duijnhoven, Iris M. C. van der Ploeg

**Affiliations:** 1 Department of Surgical Oncology, Netherlands Cancer Institute – Antoni van Leeuwenhoek, Plesmanlaan 121, NL-1066 CX Amsterdam, The Netherlands; 2 Department of Nuclear Medicine, Netherlands Cancer Institute – Antoni van Leeuwenhoek, Amsterdam, The Netherlands; 3 Department of Radiology, Netherlands Cancer Institute – Antoni van Leeuwenhoek, Amsterdam, The Netherlands; 4 Department of Radiation Oncology, Netherlands Cancer Institute-Antoni Van Leeuwenhoek, Amsterdam, The Netherlands; 5 Department of Radiation Oncology, Leiden University Medical Center, Leiden, The Netherlands; 6 Department of Surgery, Amsterdam UMC, Amsterdam, The Netherlands

**Keywords:** Breast cancer, Neoadjuvant chemotherapy, FDG-PET/CT, Incidental findings

## Abstract

**Purpose:**

Breast cancer (BC) patients undergoing FDG-PET/CT scans for neoadjuvant chemotherapy (NAC) may have additional non-BC related findings. The aim of this study is to describe the clinical implications of these findings.

**Methods:**

We included BC patients who underwent an FDG-PET/CT scan in our institute between 2011–2020 prior to NAC. We focused on patients with an additional non-BC related finding (i.e. BC metastases were excluded) for which diagnostic work-up was performed. Information about the diagnostic work-up and the clinical consequences was retrospectively gathered. A revision of all FDG-PET/CT scans was conducted by an independent physician to assess the suspicion level of the additional findings.

**Results:**

Of the 1337 patients who underwent FDG-PET/CT, 202 patients (15%) had an non-BC related additional finding for which diagnostic work-up was conducted, resulting in 318 examinations during the first year. The non-BC related findings were mostly detected in the endocrine region (26%), gastro-intestinal region (16%), or the lungs (15%). Seventeen patients (17/202: 8%, 17/1337: 1.3%) had a second primary malignancy. Only 8 patients (8/202: 4%, 8/1337: 0.6%) had a finding that was considered more prognosis-determining than their BC disease. When revising all FDG-PET/CT scans, 57 (202/57: 28%) of the patients had an additional finding categorized as low suspicious, suggesting no indication for diagnostic work-up.

**Conclusion:**

FDG-PET/CT scans used for dissemination imaging in BC patients detect a high number of non-BC related additional findings, often clinically irrelevant and causing a large amount of unnecessary work-up. However, in 8% of the patients undergoing diagnostic work-up for an additional finding, a second primary malignancy was detected, warranting diagnostic attention in selected patients.

**Supplementary Information:**

The online version contains supplementary material available at 10.1007/s10549-024-07331-9.

## Introduction

Breast cancer (BC) is the most common type of cancer worldwide, contributing to 12.5% of the total number of new cancer cases in 2020 [1]. An increasing number of patients with BC is treated with neoadjuvant chemotherapy (NAC) [2–5]. Accurate staging prior to neoadjuvant treatment is essential as it determines the appropriate overall treatment plan.

Since 2012, the Dutch Breast Cancer Guideline recommends staging by FDG-PET/CT for patients with BC with tumors larger than 5 cm (cT3), and/or positive lymph nodes (cN +) [6]. Guidelines of the European Commission Initiative on Breast Cancer (ECIBC) recommend FDG-PET/CT in patients with stage 3 BC [7, 8]. These recommendations align with NCCN guidelines, advocating against the use of FDG-PET/CT for patients with stage I, II or operable III BC [9]. In our hospital, all patients with BC eligible for NAC undergo FDG-PET/CT.

FDG-PET/CT offers advantages over conventional imaging (e.g. bone scintigraphy, ultrasound of the liver, and chest radiography), such as a higher sensitivity for staging regional lymph node involvement and distant metastases [10–13]. However, incidental findings are reported in patients with cancer undergoing FDG-PET/CT in up to 75% of the patients [14–17]. Such findings can result in additional and sometimes invasive diagnostic tests and thus more and potentially unnecessary costs and burden for the patient [14].

Patients with stage I-III BC in the Netherlands have an excellent or good long-term prognosis and, based on their subtype, are often treated with NAC [4, 18]. Patients eligible for NAC are often of young age and have a good performance status [5, 19]. Given the curative intent of treatment in this young and fit population, clinicians might tend to pursue incidental findings and therefore initiate further work-up. Current literature about incidental findings in patients with BC undergoing FDG-PET/CT reports incidental findings in 20–56% of the patients, depending on definition of incidental finding (i.e. lesions suspected for second malignancy with indication for work-up or all FDG-avid lesions) [20–22]. However, limited information exists about number and type of additional examinations and their clinical implication [21, 22].

The aim of this study is to fill this lack of information in patients with BC who undergo FDG-PET/CT prior to NAC.

## Methods

### Patient selection

All patients with BC scheduled for NAC and undergoing FDG-PET/CT prior to NAC at the Netherlands Cancer Institute Antoni van Leeuwenhoek (NKI-AvL) between January 2011 and December 2020 were included in this study. Patients with recurrent BC, those who had breast or axilla surgery prior to FDG-PET/CT, and those who did not undergo imaging of the breast (mammography, ultrasound, and/or MRI) were excluded. Eligibility for NAC was determined using (inter)national guidelines incorporating information on age, clinical stage, tumor size, and histological type. FDG-PET/CT scan was performed following standard protocol. Approximately 1 h after administration of 18F-FDG, FDG-PET/CT scanning (Philips Gemini TF Big Bore, Cleveland, OH, USA) was performed from the base of the skull to the groin region. All FDG-PET/CT reports and patient records were retrospectively reviewed for additional findings by two independent physicians (J.P.O & A.M.S.). Additional findings on FDG-PET/CT were defined as findings reported by a nuclear physician as non-physiological and undetected by breast imaging. Information about final diagnosis of the additional finding was obtained. If the diagnosis was BC related (i.e. metastases) patients were excluded. If the diagnosis was not related to BC, it was defined as a non-BC-related additional finding. Eventually, only the patients who underwent further diagnostic work-up for their non-BC-related additional finding were included.

### Data collection

Information about site, diagnostic work-up, and management of the non-BC additional findings was retrospectively collected. The different sites of additional findings were divided in lung, liver, bone, distant lymph nodes, the gastro-intestinal region (esophagus, stomach, bowel), the urogenital region (ovaries, uterus, bladder, kidney), the endocrine region (thyroid, pancreas, adrenal gland), and other (contralateral mamma, nervous system, nasopharynx and parotis). Diagnostic work-up was defined as the examinations in the first year after FDG-PET/CT. The type of diagnostic work-up that was performed included ultrasound, CT, MRI, any type of imaging-guided biopsy, endoscopy, or an endoscopy combined with a biopsy. Endoscopic procedures included gastroscopy, colonoscopy, transvaginal ultrasound, or cystoscopy. To calculate the number of examinations performed in total, the combination of imaging plus biopsy and endoscopy plus biopsy were both considered as one examination. Imaging only (Ultrasound, CT, and MRI) was scored as a non-invasive examination and imaging plus biopsy, endoscopy, or endoscopy plus biopsy as invasive examinations. Diagnoses were categorized as either normal (i.e. no abnormality on diagnostic work-up, artefact), benign, or malignant. In case of multiple diagnoses in one patient, the diagnosis with the estimated worst prognosis was scored. Management of incidental findings was classified as treatment, follow-up, or neither treatment or follow up. After one-year follow-up, changes in the diagnosis were assessed to ensure the absence of overlooked BC malignancies or second primary malignancies.

To assess the retrospective suspicion of the non-BC-related additional findings, a revision by a nuclear physician (M.P.M.S.) on all FDG-PET/CT scans with a non-BC additional finding was conducted. The purpose of this evaluation was to provide a second opinion regarding the presence of lesions that require further work-up, i.e. lesions suspected for BC metastases or a second primary malignancy. The revising nuclear physician conducted this evaluation without knowledge of the FDG-PET/CT report or the final work-up results of the additional finding, but was informed about patient characteristics and clinical BC status prior to FDG-PET/CT. The nuclear physician re-evaluated all additional findings and classified them as either intermediate or high suspicious for BC-related or secondary malignancy, indicating a need for further work-up, or low suspicious, suggesting no further work-up was indicated.

### Statistical analysis

Descriptive analyses of categorical data (frequency and percentages) and continuous data (mean and standard deviation or median and range) were calculated using SPSS, version 27 (IBM Corp., Armonk, N.Y., USA).

### Ethics approval

The study was approved by the Institutional Review Board of the Netherlands Cancer Institute Antoni van Leeuwenhoek.

## Results

### Patient selection

**Fig. 1:**
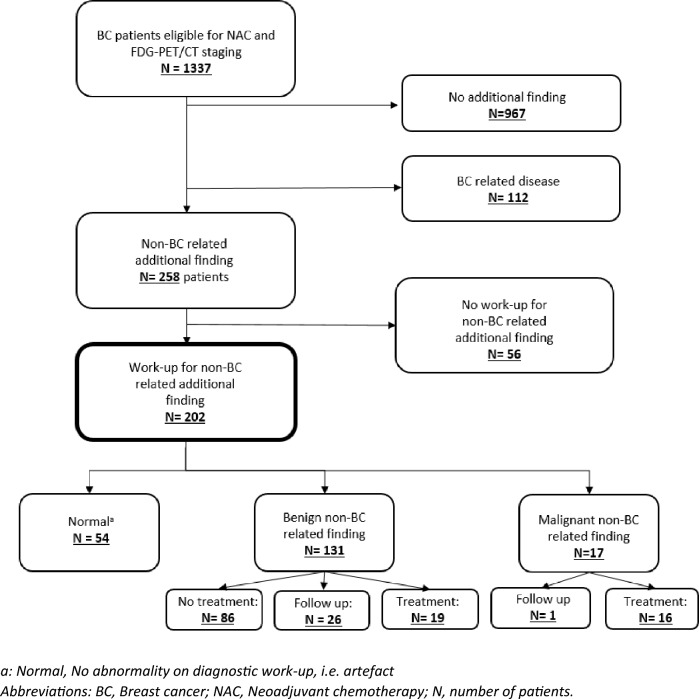
Selection of the study population of 202 patients with a non-BC-related additional finding on FDG-PET/CT and their diagnoses and management. a: Normal, No abnormality on diagnostic work-up, i.e. artefact. Abbreviations: *BC* Breast cancer, *NAC* Neoadjuvant chemotherapy, *N* number of patients

In total, 1337 patients underwent a FDG-PET/CT prior to NAC (Fig. 1). In 370 (28%) of these patients an additional finding was found on FDG-PET/CT. In 112 (30%) patients, this finding was BC related (i.e. BC metastasis) and 258 (70%) patients had a non-BC related additional finding. In 56 of the 258 patients (22%) the non-BC related additional finding mentioned in the FDG-PET-CT report was not pursued for further work-up. Of these findings, 49 (88%) were characterized as benign based on the FDG-PET/CT. In 2 patients (3%) no diagnostic work-up was performed due to progressive BC. In 5 cases (9%) there was an indication for diagnostic work-up, however, this was not performed for reasons unknown by the treating physician. Ultimately, our study included 202 patients (202/1337, 15%) with a non-BC-related additional finding who underwent diagnostic work-up.

### Patient characteristics

In the group of 202 patients who underwent diagnostic work-up, 200 patients (99%) were female, the median age was 50.5 years (IQR 44–59), 156 patients (77%) had a tumor < 5 cm (cT1-2), and 103 patients (51%) had clinically involved axillary lymph nodes (cN +).

### Diagnosis and localization of additional non-BC related finding

**Fig. 2:**
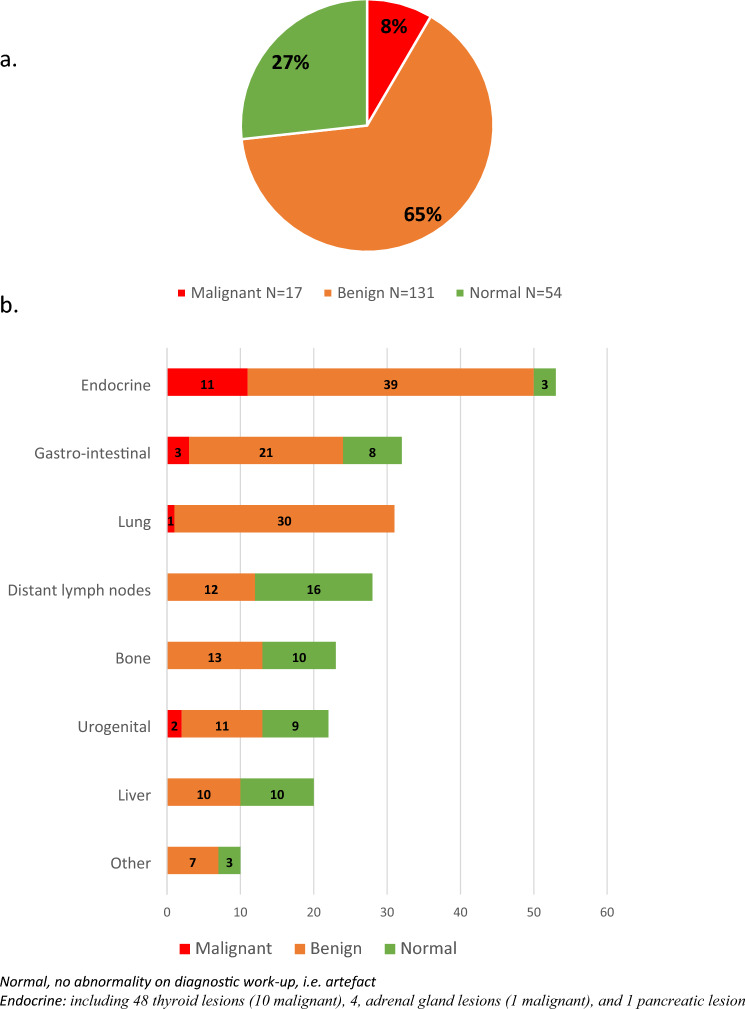
Distribution of diagnoses after work-up in 202 patients with a non-breast cancer-related additional finding on FDG-PET/CT (**a**) and localization of 219 sites affected with a non-breast cancer-related additional finding in 202 patients (**b**)

No trend over time was observed in the amount of additional non-BC-related findings for which diagnostic work-up was performed, with a median of 20 incidental findings per year. In total, 219 sites with an additional non-BC related finding underwent diagnostic work-up in 202 patients. The majority of the patients (92%) had only one site affected. Figure 2 illustrates the final diagnosis and localization. The non-BC-related findings were mostly detected in the endocrine region (53 patients, 26%: including 48 thyroid lesions, 4, adrenal gland lesions, and 1 pancreatic lesion), gastro-intestinal region (32 patients, 16%) or the lungs (31 patients, 15%) (Fig. 2b). After diagnostic work-up, 54 patients (27%) had no abnormality (i.e. normal) and 131 patients (65%) had a benign finding (Fig. 2a). Among the patients with a benign finding, 3 patients underwent surgery for a benign thyroid lesion, 2 patients underwent surgery for an ovarian cyst, and 14 patients underwent a polypectomy for an adenoma of the colon (**supplementary data**). All other benign findings did not require treatment.

Only 17 patients (8%) had a malignant finding (Fig. 2a), including 9 papillary thyroid carcinomas, 2 ovarian carcinomas, 2 colorectal carcinomas, 1 Vater papilla carcinoma, 1 medullary thyroid carcinoma, 1 adrenal carcinoma, and 1 lung carcinoma (**supplementary data**). All patients with a malignant finding were managed with curative surgery or radiotherapy, except one patient with a papillary thyroid carcinoma who opted for active surveillance (**supplementary data**).

The 9 patients with a papillary thyroid carcinoma had an excellent estimated overall survival, therefore, these malignant incidental FDG-PET/CT findings were considered prognostically secondary to their current BC. In only 8 patients (4%) the malignant additional finding was considered more decisive for survival than the diagnosed BC.

Overall, in the 1337 patients eligible for NAC, only 17 (1.3%) had a second primary malignancy of which 8 (0.6%) were more prognosis determining than their current BC.

### Diagnostic work-up

**Fig. 3:**
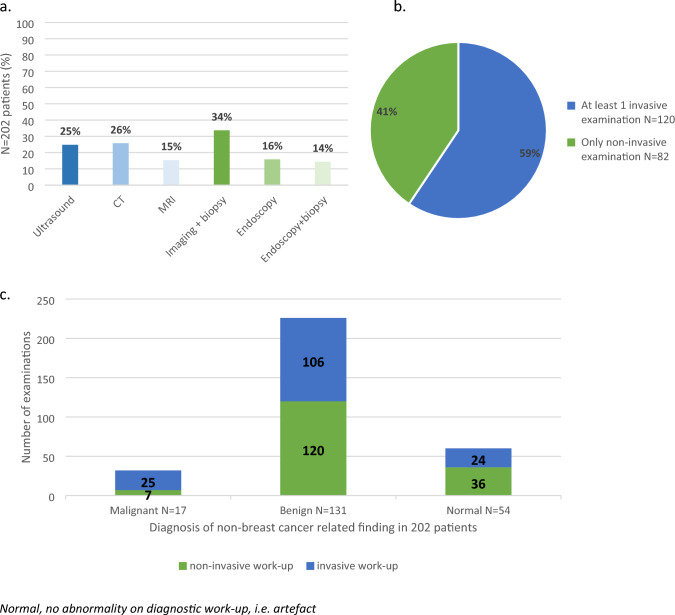
The characteristics of the 318 work-up examinations performed in 202 patients with a non-BC-related additional finding. The different types of work-up examinations per patient (**a**), the number of patients who underwent at least 1 invasive examination (**b**), and the number of invasive/non-invasive examinations per diagnosis in 202 patients (**c**)

In the first year, after the FDG-PET/CT scans, a total of 318 examinations were conducted in 202 patients. Figure 3a presents the type of examinations per patient. The type of test performed in most patients was imaging + biopsy (35%), followed by CT scan (26%) and ultrasound (25%). Figure 3b shows that 120 patients (59%) underwent at least 1 invasive examination. As displayed in Fig. 3c, the patients with a malignant diagnosis underwent a higher percentage (25/32, 78%) of invasive examinations compared to patients with a benign (106/236, 47%) or normal (24/60, 40%) finding.

### Independent revision of the FDG-PET/CT scans

**Fig. 4:**
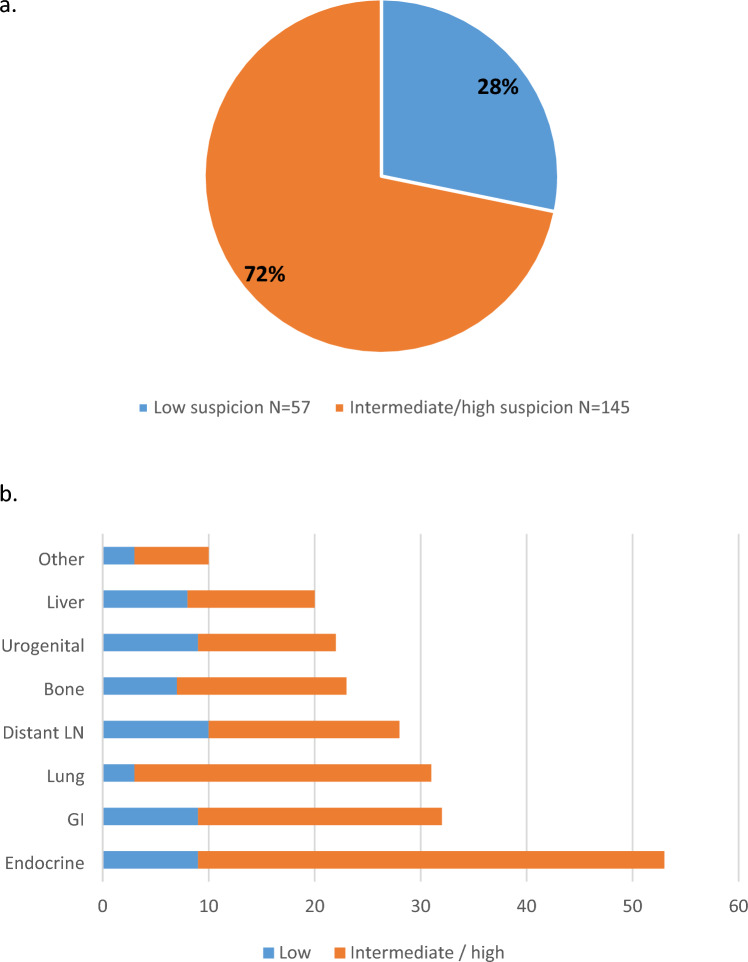
The level of suspicion of the non-breast cancer-related findings on FDG-PET/CT after revision of a nuclear physician. The percentage of the 202 patients with an intermediate or high suspicious additional finding against the patients with at least one low-suspicious additional finding for which no diagnostic work-up was advised (**a**), categorized for the 219 affected sites (**b**)

**Fig. 5:**
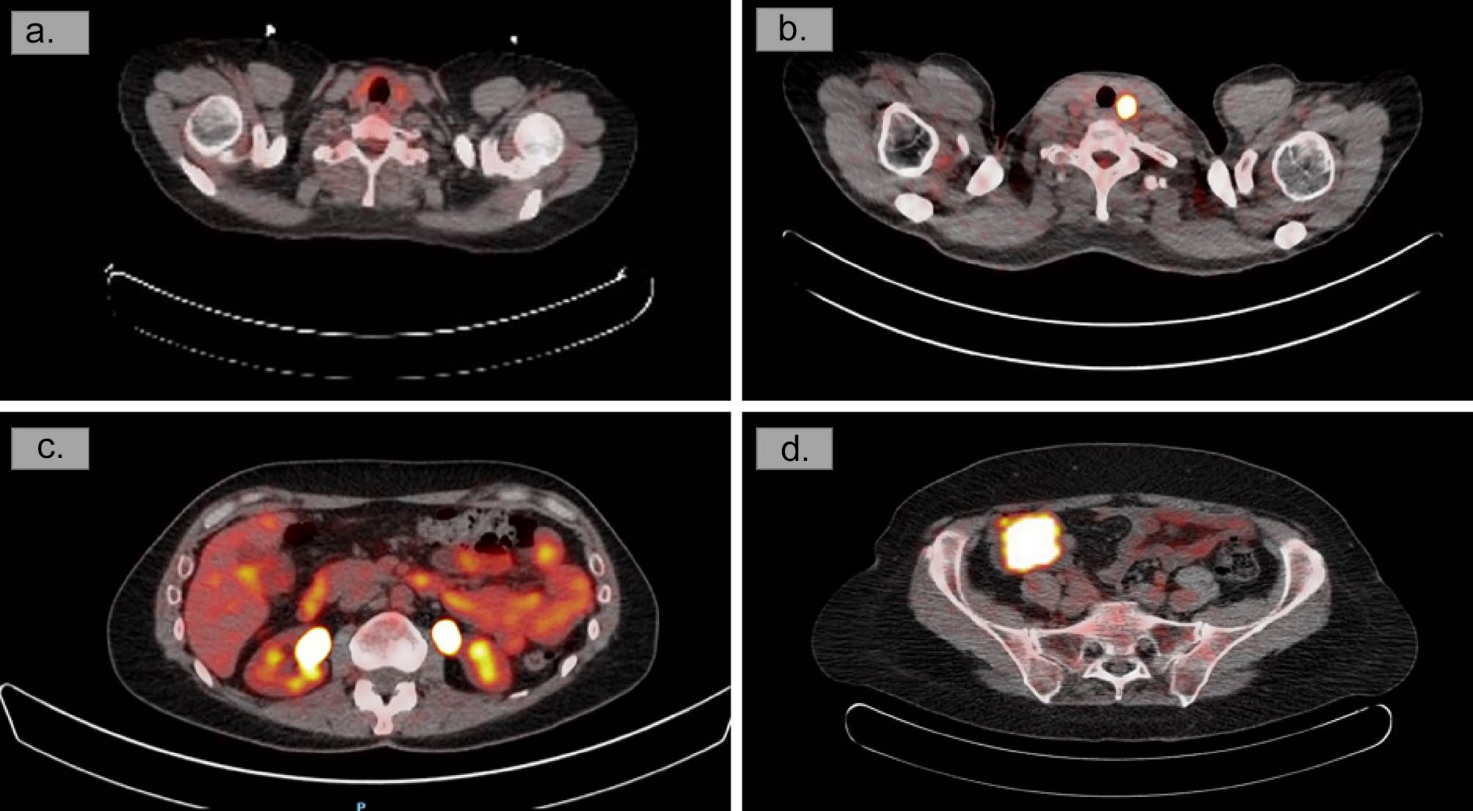
Malignant and benign additional non-BC-related findings. A benign multinodular thyroid lesion (**a**) compared to a papillary thyroid carcinoma with focal uptake (**b**) and a gastro-intestinal lesion with diffuse FDG uptake without any abnormality on colonoscopy (**c**) compared to a colon carcinoma (**d**)

The revision process by the independent nuclear physician assessed that in 57 out of 202 patients (28%) who underwent diagnostic work-up, at least one lesion did not require further investigation in retrospect. Figure 4 shows the distribution of the low-suspicion additional findings across the various sites, each representing a small percentage of the total. Figure 5 shows 2 examples of high- and low-suspected lesions.

## Discussion

This study investigated the clinical implications of non-BC-related additional findings on FDG-PET/CT in patients eligible for NAC. We observed that 28% (370/1337) of the patients had additional findings, of which 202 patients (15%) had a non-BC-related finding resulting in a total of 318 extra diagnostic examinations. In over half of these 202 patients an invasive diagnostic procedure was performed during work-up; imaging plus biopsy (34%), endoscopy (15%), and endoscopy plus biopsy (14%). Ultimately, only a small number of 17 (8%) patients had a second primary malignancy, and 8 (4%) of these additional malignancies were considered to have an estimated worse prognosis compared to the earlier diagnosed BC. In the whole population of 1337 patients, 1.3% had a second primary malignancy and 0.6% a more prognosis determining diagnosis than their current BC.

It is widely recognized that FDG-PET/CT frequently reveals incidental findings. The incidence of these findings depends on patient population and the definition of an incidental finding [14–17, 20]. Older patients and patients with malignancies tend to have a higher number of incidental findings on FDG-PET/CT [14]. Variations in the definition of incidental findings across studies is seen, as some studies report all additional findings, while others focus only on suspected incidental findings. The incidence rates in these reports range from 12–75%, in this study in 28% of the patients an additional finding was reported [14, 15]. The decision to further evaluate an incidental finding depends on patient-specific factors, the level of suspicion associated with the finding and the treating physician’s assessment. In a study conducted by Srour et al., it was reported that 146 out of 262 (55.7%) patients who underwent FDG-PET-CT prior to NAC had incidental findings [20]. This study included patients with findings deemed “likely” to be physiological, and no further work-up was outlined for these findings. Moreover, Vogsen et al. conducted a study involving 103 patients with high-risk primary BC undergoing FDG-PET/CT, resulting in 24 (23%) patients undergoing examinations for incidental findings [21]. In a separate prospective cohort of 225 patients with suspected recurrent BC undergoing FDG-PET/CT, Vogsen et al. observed incidental findings necessitating further examinations in 46 patients (20%) [22]. These results align with the 15% incidence observed our study. There is currently a lack of national or international guidelines addressing the management of additional findings on FDG-PET/CT scans.

To gain better insight on the current work-up of additional findings on FDG-PET/CT, we specifically focused on patients who had a non-BC-related finding that was deemed non-physiological and who underwent additional diagnostic work-up. In this group a high number of diagnostic work-up was performed (318 examination sin 202 patients), resulting in the detection of only 17 (17/202 = 8% or 17/1337 = 1.3%) new malignancies, which seems to align with previous literature. In two separate studies conducted by Beatty et al. and Hadad et al., cancer patients who underwent a FDG-PET/CT were found to have a second primary malignancy in 1.8% and 0.6% of cases, respectively [15, 16]. Vogsen et al. reported a second primary malignancy in 3.8% of high-risk BC patients undergoing FDG-PET/CT.[21]Notably, 10 patients in this study were diagnosed with thyroid cancer, which is a frequently reported incidental finding on FDG-PET/CT scans [23]. A correlation between BC and thyroid cancer has been reported; thyroid cancer occurs at an incidence of 0.1% among patients with BC [24]. In our study, we observed thyroid cancer in 0.74% of the patients, consistent with the existing literature. Most of these cases were papillary thyroid carcinomas (9/10, 90%), which has a favorable prognosis with a 10-years overall survival of 97% [25]. Therefore, we considered a papillary thyroid carcinoma not to be prognosis-determining.

In general, to minimize unnecessary work-up, nuclear medicine physicians consider various variables (such as BC stadium, localization of the incidental finding and the pattern of FDG uptake) to assess whether a lesion is suspected for a second primary malignancy or BC-related metastases. Advanced BC, high-FDG uptake in the primary tumor, focal aspect of FDG uptake, and high SUVmax and/or SUVpeak are associated with a higher likelihood of a (BC-related) malignancy. [17] Additionally, different sites necessitate different work-up strategies [17]. Nevertheless, our study reveals a persistently high rate of unnecessary diagnostic work-up procedures. During the second review of all FDG-PET/CT scans conducted by a nuclear physician, we found that 28% of our study patients who had undergone work-up for an initially reported non-BC-related additional finding, had no indication for further work-up in retrospect. In the group of additional endocrine findings, 9 out of 46 (20%) thyroid glands showed diffuse uptake. These lesions were not suspected by the second reviewer and none of these lesions turned out to be a malignancy. It is already known that these non-focal uptake sites are mostly physiological of benign and therefore not necessary to be pursued by additional work-up [26]. Furthermore, we discovered that 24 of the 31 initially reported lung nodules (77%) were non-specific millimetric lung nodules lacking FDG-uptake that, in hindsight, do not need further work-up. We suggest that all additional lung findings warrant a routine thorough scoring assessment using the Fleischner criteria to avoid unnecessary follow-up [27]. Additionally, we found that distant lymph nodes were investigated even in the absence of suspected axillary lymph nodes or the presence of an obvious infectious focus. In such cases, these lesions are more likely to be considered as physiological or benign. Similarly, in the case of FDG uptake in the urogenital region, which is often influenced by hormonal fluctuations, these lesions may also be more frequently regarded as physiological. However, caution is needed in patients with a BRCA genetic mutation or in patients of older age. Finally, diffuse uptake in gastrointestinal and liver lesions should also mostly be interpreted as unsuspected. It is recommended to explicitly report these findings as physiological or benign. By addressing these specific findings and highlighting their unsuspected nature, we can avoid unnecessary investigations and provide more accurate guidance for clinical decision-making.

It should be noted that FDG-PET/CT offers many advantages. In the regional setting, FDG-PET/CT demonstrates high sensitivity for detecting regional metastases, resulting in changes of locoregional treatment (i.e. surgery and/or radiotherapy) [10, 28, 29]. Furthermore, FDG-PET/CT is superior to conventional imaging in detecting distant metastases, influencing treatment decisions [11, 12]. In this study BC-related findings, i.e. metastases are excluded. In this cohort 112/1337 patients eventually had distant metastasis on FDG-PET/CT [11]. We found a low incidence of non-BC related additional findings in the bone, liver, and distant lymph nodes. However, since these organs are known to be preferred metastases sites, it is premature to conclude that additional findings in these locations do not necessitate further investigation in all cases [30].

This study has certain limitations that should be acknowledged. First, it was conducted at a single tertiary cancer center, which may limit the transferability of the findings to other hospitals. Furthermore, it is important to acknowledge that our study primarily relies on retrospectively gathered information extracted from FDG-PET/CT reports. For inclusion in the study, the patients needed to have an additional finding mentioned in the report followed by diagnostic work-up. It is important to note that these reports may be influenced by the subjectivity and variability of the reporting nuclear physician, as different physicians might interpret and report findings differently. Additionally, we cannot be certain whether the nuclear physician intended to advise diagnostic work-up solely by mentioning the finding in the FDG-PET/CT report, because often there was no level of suspicion reported. However, as a result of this, our study provides valuable insight into daily clinical practice. Furthermore, it is important to acknowledge the potential for slight bias in the revising nuclear physician’s evaluation, given that only the 202 FDG-PET/CT scans with additional non-BC-related additional findings were reviewed. While the revising nuclear physician was blinded for the initial reports and the final diagnosis, the selective review of scans could introduce a subtle bias. Additionally, considering inter-rater variability, the revising nuclear physician was very experienced, whereas the initial reports were authored by different nuclear physicians with varying levels of experience. This diversity in experience might have contributed to the variability in the interpretation of the non-BC-related findings across the FDG-PET/CT scans.

In conclusion, this study underscores the high rate of additional invasive diagnostic examinations performed for non-BC-related FDG-PET/CT findings, resulting in only a limited number of clinically relevant findings. In case of performing FDG-PET/CT it is important to balance out the advantages of improved staging and early detection of secondary primary malignancies in 1.3% of patients with the disadvantages of invasive work-up for irrelevant non-BC-related findings, challenging logistics and costs. Besides further specifying the need for FDG-PET/CT in patients with BC eligible for NAC, physicians must be aware of the low-clinical relevance of non-BC-related additional findings. Still, it must be kept in mind that there is a slight change of early detection of a second primary malignancies with good treatment options. We recommend a multidisciplinary approach to determine the need for further work-up of all FDG-PET/CT additional lesions and to limit the amount of invasive procedures with an expected low yield for relevant findings. Future studies about cost-effectiveness of FDG-PET/CT in patients with BC eligible for NAC or the implementation of artificial intelligence to determine which non-BC-related findings need diagnostic work-up would ideally lead to an unambiguous guideline.

### Supplementary Information

Below is the link to the electronic supplementary material.

Supplementary file1 (DOCX 26 KB)

## Data Availability

The datasets are available from the corresponding author on reasonable request.

## Abbreviations

BC: Breast cancer
FDG-PET/CT: Fluorodeoxyglucose-positron emission tomography / computed tomography
NAC: Neoadjuvant chemotherapy
SUV: Standard uptake value
