# Supplementary material for: Clinical implications of non-breast cancer related findings on FDG-PET/CT scan prior to neoadjuvant chemotherapy in patients with breast cancer
Source: Breast Cancer Res Treat. 2024 Jun 12;206(3):585–94. doi: 10.1007/s10549-024-07331-9 (PMC11208275; doi:10.1007/s10549-024-07331-9)
Supplement: Supplementary file 1 — Supplementary file1 (DOCX 26 KB) [file 10549_2024_7331_MOESM1_ESM.docx]

| **Supplementary data.** All non-breast cancer related findings: diagnosis and management | | | |
| --- | --- | --- | --- |
| **Site** | **Diagnosis^a^** |  | **Management** |
| **Endocrine**  (N=53) | **11** malignant | **1** adrenal carcinoma  **1** medullary thyroid carcinoma **9** papillary thyroid carcinoma^b^ | transabdominal extirpation + chemotherapy thyroidectomy  **8** (hemi)thyroidectomy **1** follow up |
|  | **39** benign | **36** benign thyroid lesion   **3** benign adrenal lesion | **3** surgery **13** follow up **20** no treatment **2** no treatment **1** Follow up |
|  | **3** normal |  |  |
| **Gastro-intestinal**  (N=32) | **3** malignant | **1** colon carcinoma  **1** rectal carcinoma  **1** Vater papilla carcinoma | hemicolectomy low anterior resection + radiotherapy Whipple |
|  | **21** benign | **14** colon: tubular adenoma  **3** gastritis  **2** infectious **1** anal fissure **1** normal anastomosis | **14** polypectomy  **3** no treatment **2** no treatment **1** no treatment **1** no treatment |
|  | **8** normal |  |  |
| **Lung**  (N=31) | **1** malignant | **1** pulmonary carcinoma | radiotherapy |
|  | **30** benign | **6** infectious  **24** non-specific lung nodule | **3** follow up **3** no treatment **4** follow up **20** no treatment |
| **Distant lymph nodes**  (N=28) | **12** benign | **12** reactive/infectious | **12** no treatment |
|  | **12** normal |  |  |
| **Bone**  (N=23) | **13** benign | **4** hemangioma **9** benign bone lesion | **4** no treatment **1** follow up **8** no treatment |
|  | **10** normal |  |  |
| **Urogenital**  (N=22) | **2** malignant | **2** ovary carcinoma | **2** transabdominal extirpation |
|  | **11** benign | **5** benign adnexal cyst  **6** uterine myoma | **2** diagnostic surgery **3** no treatment **6** no treatment |
|  | **9** normal |  |  |
| **Liver**  (N=20) | **10** benign | **6** hemangioma  **2** adenoma **2** posttraumatic | **2** follow up **4** no treatment **2** no treatment **1** follow up **1** no treatment |
|  | **10** normal |  |  |
| **Other**  (N=10) | **7** benign | **3** contralateral mamma:   benign lesion **2** schwannoom  **1** parotis: pleomorphic adenoma **1** benign nasopharyngeal tumor | **1** follow up **2** no treatment **1** follow up **1** no treatment **1** follow up  **1** follow up |
|  | **3** normal |  |  |

*a: Normal = no abnormality on diagnostic work-up, i.e. artefact
b: Malignancies considered less prognosis determing than the current breast cancer*
